# Construction and characterization of a bacterial artificial chromosome library for *Gossypium mustelinum*

**DOI:** 10.1371/journal.pone.0196847

**Published:** 2018-05-17

**Authors:** Yuling Liu, Baohong Zhang, Xinpeng Wen, Shulin Zhang, Yangyang Wei, Quanwei Lu, Zhen Liu, Kunbo Wang, Fang Liu, Renhai Peng

**Affiliations:** 1 Anyang Institute of Technology, Anyang, Henan, China; 2 Department of Biology, East Carolina University, Greenville, NC, United States of America; 3 State Key Laboratory of Cotton Biology / Institute of Cotton Research of Chinese Academy of Agricultural Science, Anyang, Henan, China; USDA-ARS Southern Regional Research Center, UNITED STATES

## Abstract

A bacterial artificial chromosome (BAC) library for *G*. *mustelinum* Miers ex G. Watt (AD_4_) was constructed. Intact nuclei from *G*. *mustelinum* (AD_4_) were used to isolate high molecular weight DNA, which was partially cleaved with *Hind* III and cloned into pSMART BAC (*Hind* III) vectors. The BAC library consisted of 208,182 clones arrayed in 542 384-microtiter plates, with an average insert size of 121.72 kb ranging from 100 to 150 kb. About 2% of the clones did not contain inserts. Based on an estimated genome size of 2372 Mb for *G*. *mustelinum*, the BAC library was estimated to have a total coverage of 10.50 × genome equivalents. The high capacity library of *G*. *mustelinum* will serve as a giant gene resource for map-based cloning of quantitative trait loci or genes associated with important agronomic traits or resistance to *Verticillium* wilt, physical mapping and comparative genome analysis.

## Introduction

*Gossypium* genus comprises more than 50 species, including eight diploid groups and six tetraploid species [1–3]. Among the six tetraploid species, *G*. *hirsutum* L. (AD_1_) and *G*. *barbadense* L. (AD_2_) have been domesticated. The remaining tetraploid species are all wild species, and have much narrower distributions: *G*. *tomentosum* Nuttall ex Seemann (AD_3_) in the Hawaiian Islands, and *G*. *mustelinum* Miers ex Watt (AD_4_) in NE Brazil, *G*. *darwinii* Watt (AD_5_) in the Galapagos Islands[4], except for *G*. *ekmanianum* Wittmack (AD_6_), whose distribution needs to be further determined[3, 5]. Because of long-term natural selection under the distrubution environment, these wild species have rich genetic diversity and contain many excellent genes which can be used in cotton breeding, such as drought resistance, disease resistance, cold resistance, and the potential properties of fine fibers [6]. Phylogenetic and phenetic ananlyses demonstrate that *G*. *mustelinum* had been isolated as one branch of the earliest split following allopolyploid formation, and is genetically farthest from *G*. *hirsutum* and other tetraploid species [4, 5, 7]. So most likely there have some new genetic loci that can be used for genetic improvement of upland cotton, which is helpful to solve the serious bottleneck of genetic diversity in the development of upland cotton varieties [8–10]. Moreover, *G*. *mustelinum* showed a special chromosome structure based on the study of molecular cytogenetics [11]. As a very special tetraploid wild cotton species, *G*. *mustelinum* has irreplaceable value in basic research and germplasm. However, there are few reports on its genome research because of the difficulty to obtain experimental material and complexity of genome [12].

Bacterial artificial chromosome (BAC) libraries carrying large insert genomic DNA, are essential genomic resources for map-based gene cloning and and physical map construction [13–15], development of markers based on BAC-end sequences [16, 17], genome sequencing of complex genomes [18]. So far, dozens of BAC libraries have been reported for different *Gossypium* species. Such as libraries from *G*. *hirsutum* [19–23], *G*. *barbadense* [24], *G*. *tomentosum* [25], *G*. *arboretum* [26], *G*. *herbaceum* var. *africanum* [27], and *G*. *raimondii* [28]. Based on these resources, researches on evolutionary relationship of *Gossypium* [29], individual chromosome identification and naming [30, 31], physical map construction and guidance in genome sequence assembly [32, 33] have been carried out.

In this paper, we firstly report the construction of a high capacity library in *G*. *mustelinum*, a wild tetraploid species, which is far from *G*. *hirsutum* in phylogeny, and has many excellent traits, including finer fibers and *Verticillium* resistance, which will help to save cotton germplasm resources, and provide important genome resources for cultivated cotton breeding programs about genetic diversity.

## Materials and methods

### Plant material and high-molecular-weight (HMW) DNA preparation

Wild tetraploid cotton species, *G*. *mustelinum* (AD_4_) (accession P0811704) were conserved in the greenhouse at CRI-CAAS in Anyang City, Henan Province, China. Cotton mature plants were grown in dark for about 10 day to prepare etiolated young leaf tissues. For chromosome preparation, seeds of *G*. *mustelinum* were planted in nutrition bowls for 7 d to harvest root tip.

The etiolated young leaf tissues were sampled, then immediately frozen in liquid nitrogen. Nuclei were isolated from about 30 g etiolated young leaves, according to the procedure described by Zhang et al. [34] and Wang et al. [24]. Purified nuclei were embedded in 1% low melting point (LMP) agarose to make agarose plugs. After lysis with proteinase K, the quality of HMW DNA was tested by pulse-field gel electrophoresis (PFGE) at 6 V/cm, switch times of N/S 50 s, E/W 50 s, 11°C for 18 h.

### Generation of HMW genomic DNA fragments for cloning

The HMW DNA was digested with different *Hind* III (TaKaRa) concentrations to determine the optimum partial restriction digestion conditions for generating the highest percentage of DNA fragments between 100 and 300 kb. Every one half of the three randomly selected DNA plugs was equilibrated with restriction enzyme buffer for 30 min, then digested respectively with 0, 1, 2, 3, 4, 5, and 6 U *Hind* III in 37°C water bath for 30 min. The digested products were tested by PFGE at 6 V/cm, switch times of N/S 50 s, E/W 50s, 11°C for 18 h. Based on the optimal digestion conditions, digestion of the nucleus DNA were carried out. Then, two rounds of size selection of the partially digested products were carried out. The first size selection was aimed to recover gel fraction containing 100–300 kb fragments by PFGE at 6 V/cm, switch times of N/S 50 s, E/W 50s, 11°C for 18 h. The second size selection was performed using the first size-selected fractions cut from the center part of the gel fractions corresponding to 100–200 kb fragments and 200–300 kb fragments by PFGE at 6 V/cm, switch times of N/S 4 s, E/W 4s, 11°C for 14 h. The size-selected DNA was electroeluted in BIO-RAD PowerPac Electro-Eluter at 10 mA for 2.5 h. The concentrations of the electroeluted DNA were measured by 1% agarose gel electrophoresis (85 V, 40 min) using standard λDNA with different concentration gradient.

### Ligation and bacterial transformations

The pSMARTBAC (*Hind* III) cloning vector (Lucigen) was used for library construction. Electroeluted DNA fragments and vector (molar ratio 1:10) were ligated using T4 DNA ligase in a 50 μL reaction solution at 16°C for 16 h. Then, the reaction solutions were heated to 65°C for 10 min to inactivate ligase, nextly, were desalted and concentrated by floating on ddH_2_O/PEG8000 with 0.025 μm Millipore filters for 2 h/1 h respectively.

Total ligation product (about 1–2 μL) was transformed into 20 μL EPI300 cells (Epicentre) using a MicroPulser Electroporation Apparatus (Bio-Rad) with settings of 2.5 kV, charge time 3 ms. Next, transferred cells culturing, positive clones picking and storing were performed according to the protocol described by Wang et al. [24].

### BAC clones characterization

To estimate insert sizes, BAC clones were selected at random from the library and inoculated into 8 mL 2 × YT medium containing chloramphenicol (12.5 μg/mL) and incubated at 37°C for 14 h. BAC-DNA was isolated using Hipure BAC Mini Kit (Magen) according to the handbook. BAC-DNA (10 μL) was digested with 3 U of *Not* I enzyme for 3 h at 37°C. Insert sizes were estimated based on results of PFGE with 𝜆 PFG Ladder (NEB) as a reference.

Library stability was tested using 5 BAC clones selected at random from the library according to the protocol described by Hu et al. [26].

### BAC library screening and positive BAC clone FISH verifying

To evaluate the BAC library, the SSR marker Gh216, once used to obtain a subgenome-specific BAC clone[33] was selected to screen the library using bacteria liquid-PCR according to our protocol previously described [15]. The primers (F/R) of Gh216 (TCCACATTCCCATGCACTACTC/CTAAAACCTTATACATACAAAATGCAGC) were synthesized by Shanghai Sangon Biotech Inc. The selected positive BAC clones were cultivated with LB medium containing 12.5 μg/ml chloramphenicol for 14 h. Then BAC-DNA were isolated using Hipure BAC Mini Kit (Magen) according to the handbook and used to label probes for FISH. Chromosome preparation and FISH were carried out following previous protocol [15].

## Results

### High-molecular-weight genomic DNA preparation

**Fig 1:**
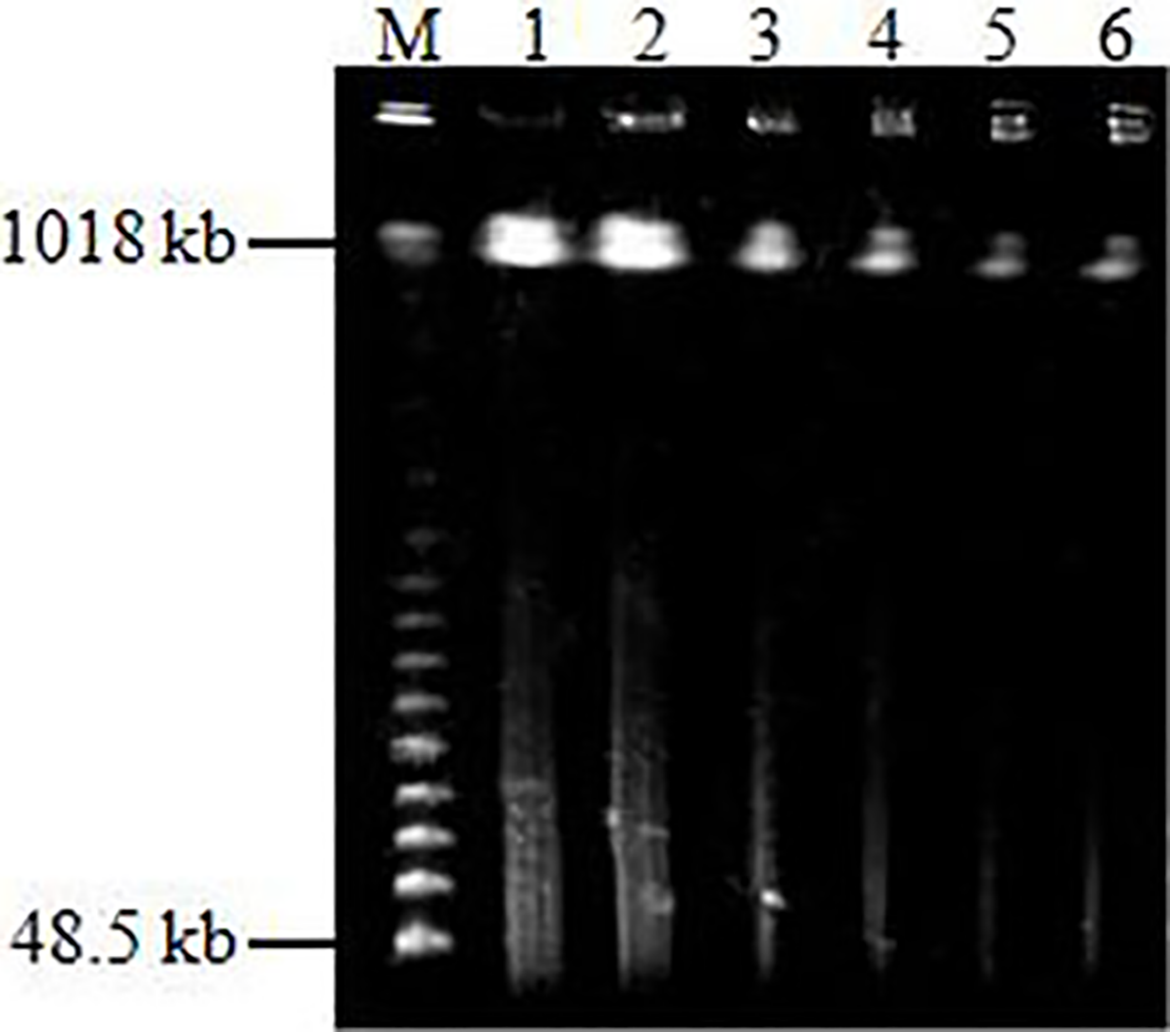
The quality of HMW DNA tested by PFGE. M, 𝜆 PFG Ladder (NEB); Lanes 1–6 were 1/2, 1/2, 1/4, 1/4, 1/8, 1/8 plug DNA.

Undigested HMW DNAs isolated from etiolated young leaves had a mean length 1000 kb, and were seldom degraded (Fig 1), which can meet the needs of library construction.

### The optimum partial restriction digestion condition and insert DNA prepration

**Fig 2:**
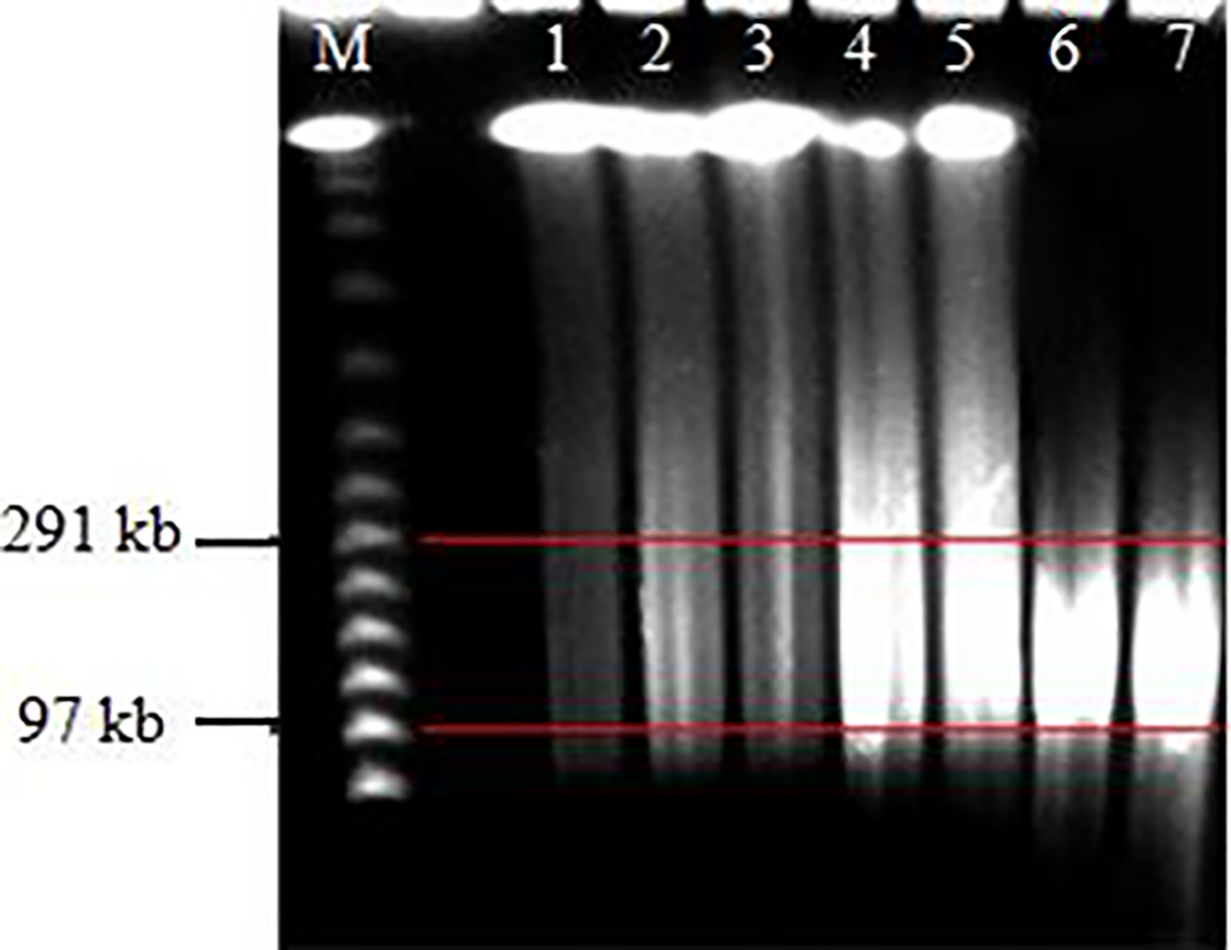
Determination of the optimized enzyme dosage for HMW DNA. M, 𝜆 PFG Ladder (NEB); 1–7 indicated 0 U, 1 U, 2 U, 3 U, 4 U, 5 U, 6 U of *Hind* III, respectively.

**Fig 3:**
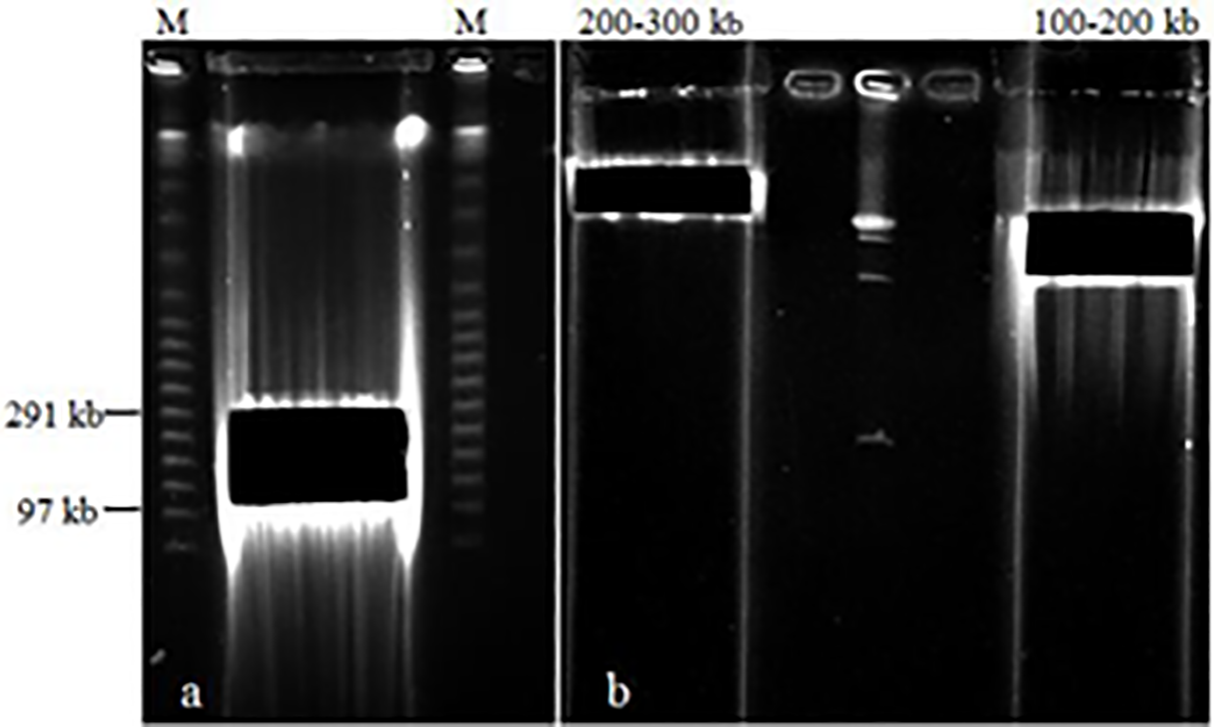
The first selection (a) and the second selection (b) restriction fragment of DNA.

**Fig 4:**
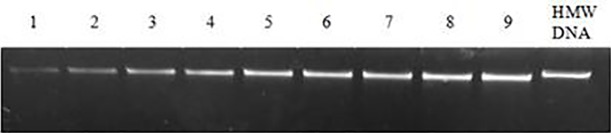
Detection of HMW DNA concentrations. Lanes 10 was HMW DNA; Lanes 1–9, λDNA concentrations were 1 ng/μL, 2 ng/μL, 3 ng/μL, 4 ng/μL, 5 ng/μL, 6 ng/μL, 7 ng/μL, 8 ng/μL, 10 ng/μL, respectively.

The suitable insert fragment length for BAC library construction is 100–300 kb. Digestion time and enzyme concentration are key factors for size control. Based on the result of PFGE, the optimal condition was digestion with 3 U *Hind* III at 37°C for 30 min, which concentrated more fragments between at 100–300 kb (Fig 2). A mass digestion was performed under the above condition to produce insert DNA. The digested DNA was treated with two rounds of electrophoresis, the fragments of 100–200 kb and 200–300 kb were recovered from the gel (Fig 3). The concentrations of the electroeluted DNA was approximately 5 ng/μL based on the detection with concentration-knownλDNA (Fig 4), which suits for the needs of connection transformation.

### BAC library construction and characterization

**Fig 5:**
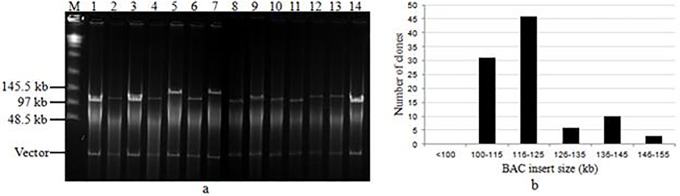
Evaluation of insert sizes of BAC clones. a, M, 𝜆 PFG Ladder (NEB); Lanes 1–14, *NotI* enzyme digestion of BAC single clones. b, insert size distribution.

The library was constructed after ligation and electroporation, which consists of 208,182 individual clones. All of the clones were handpicked and stored in 542 384-microtiter plates (Table 1). Ninty-eight BACs were randomly selected from the BAC library for size estimation. After digestion of BAC-DNA with *Not* I enzyme, the insert DNA fragment was separated from the vector (Fig 5A). Based on analysis, the average insert size of the BAC library was 121.72 kb ranging from 100 to 150 kb (Fig 5B). About 2% of the clones did not contain inserts (2 enpty clones to 98 selected clones). Based on an estimated genome size of 2372 Mb for *G*. *mustelinum* [35], the BAC library was estimated to have a total coverage of 10.50 × genome equivalents. The probability of finding any specific locus from this BAC library is estimated to be 99.9% according to the formula N = ln(1-p)/ln(1-f/G), where P is the probability, N is the number of clones, f is the average insert size of clones, and G is the haploid genome size [36].

**Table 1:** Statistics of BAC library of *G*. *mustelinum*.

Clone numbers	208,182
Vector	pSMARTBAC (*Hind* III)
Comptetent cell	EPI300 cells
Average inset size	122 kb
Haploid genome equivalent	10.50 ×
Insert-empty BACs	4,164 (2%)

**Fig 6:**
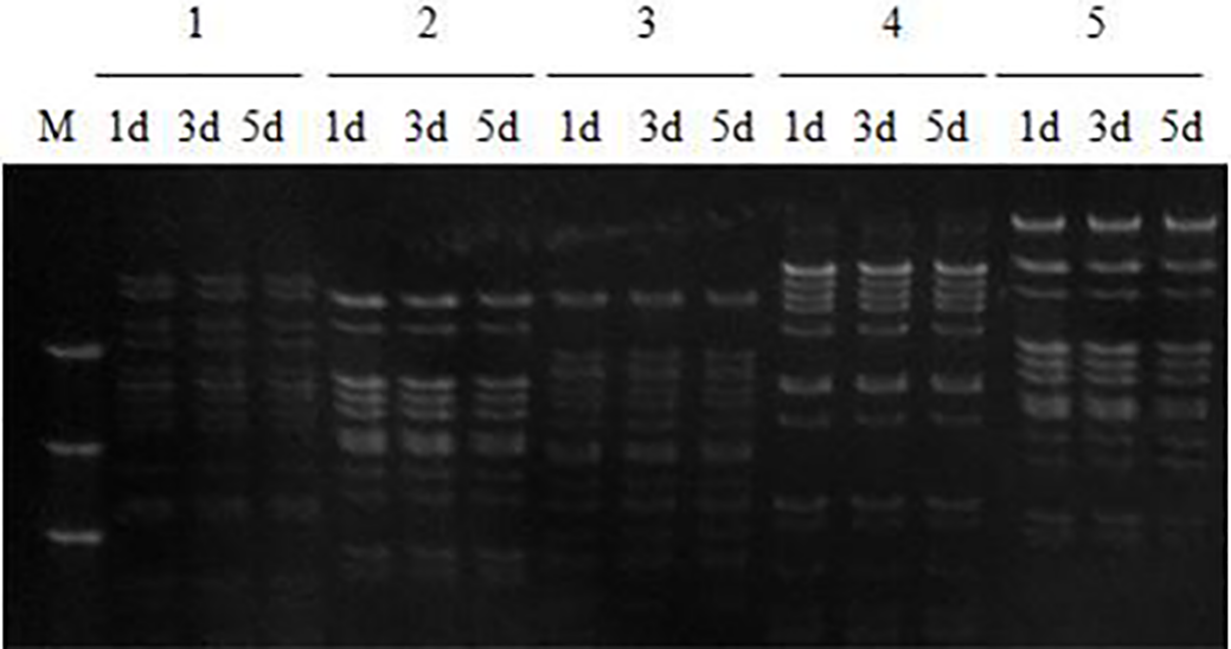
Stability analysis of five BAC clones. M, DL2000 DNA Marker; 1–5, five BAC single clones; 1d, 3d, 5d, samples from the first day, the third day and the fifth day.

We analyzed the Hind III restriction patterns of five BAC clones after culturation of 1 d, 3 d and 5 d. No visible changes were seen in fingerprints of three BAC-DNA samples of each BAC clone (Fig 6), indicating the good stability of the BAC library.

### BAC library screening and FISH verifying

**Fig 7:**
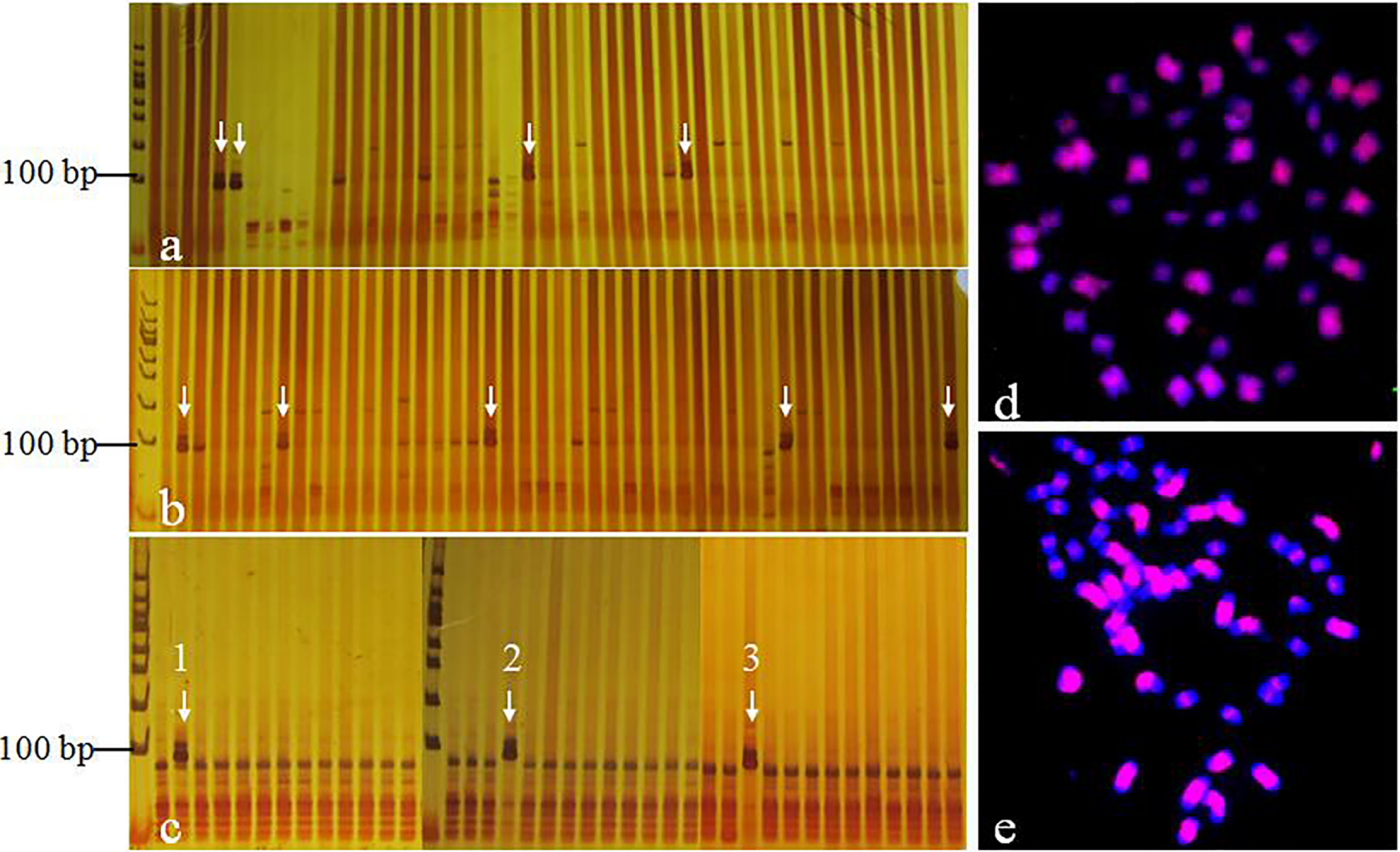
Positive BAC clones and FISH mapping. a and b, nine positive super-pools (white arrows) based PCR-screening of 96 super-pools; c, three positive BAC clones (white arrows, 1-32N22, 2-48M15, 3-89H09) from 3 of 9 super-pools; d, FISH maping of 48M15 (red) on metaphase chromosomes of *G*. *mustelinum*; e, FISH maping of 57I23 (red) on metaphase chromosomes of *G*. *mustelinum*.

For library screening, we constructed 96 super-pools containing 36,864 BAC clones. After bacteria liquid-PCR screening with Gh216 SSR primers, nine super-pools with positive bands were obtained (Fig 7A and 7B). Further PCR screening against corresponding row and column pools, three positive BAC clones for Gh216 SSR marker were identified finally (Fig 7C, Table 2). According to the formula N = ln(1-p)/ln(1-f/G), the probability of finding a specific locus from a set of 36,864 BAC clones of *G*. *mustelinum* is about 85%, which is less than the success rate of three to one. This result was consistent with previous studies about Gh216-anchored BAC clone 57I23, which enriched in repeats distinguishing subgenome of allotetraploidy cottons [33]. In order to verify genome distribution of the identified BAC clones, FISH was performed using BAC-DNA of 48M15 as probe to hybridized with metaphase chromosomes *G*. *mustelinum*. Dispersed signals on the chromosomes were viewed (Fig 7D), but with a little lower coverage relative to BAC 57I23 identified previously (Fig 7E).

**Table 2:** BAC clones screened from the BAC library.

SSR marker	Screened BAC clones
Gh216	32N22, 48M15, 89H09

## Discussion

The obtaintion of high-quality nuclear DNA is the foundation for construction of BAC library. Generally, yellowing cotyledon of seedlings were selected as the materials for DNA extraction to avoid contamination of the chloroplast DNA. As rare wild resources, *G*. *mustelinum* has fewer seeds and lower seed germination rate, yellowing cotyledons are not easy to obtain. In this paper, we chose mature plants growing perennially in greenhouse to prepare etiolated young leaf tissues by growing in dark for about 10–15 days. Pure, high quality and complete HMW-DNA suiting for construction of BAC library was obtained from the above leaf tissues.

Previous studies have shown that a clonal coverage of 6.0–8.0 genome equivalents is sufficient for coressponding research on genome-wide genetic dissection of complex organisms [20, 21, 23, 24]. Here, we have successfully constructed a *G*. *mustelinum* BAC library, which is large-insert, deep-coverage and lower empty vector rate. The high coverage BAC library contains all or most of the genomic information, which can be used for germplasm resources preservation permanently in view of the scarcity of *G*. *mustelinum* plant material [12].

In this study, the SSR marker Gh216 was used to screen the BAC library for specific BAC clones. This marker has once been used to obtain a subgenome-specific BAC clone enriched in repetitive sequences from the BAC library of *G*. *herbaceum* var. *africanum* [33]. The BAC clones identified by this marker may be candidate clones containing specific repetitive sequences. Here, using the bacteria liquid-PCR screening strategies, we identified 3 positive BAC clones from 96 super-pools containing 36,864 BAC clones, which indicate a higher positive clone screening rate from our BAC library.

At the same time, based on the constructed BAC library, studies on genome structure of *G*. *mustelinum*, including BAC-FISH-based karyotyping, physical mapping and even whole genomic sequencing, will be carried out, and all these works will serve as an important genomics resource to clone other genes conferring agronomically important traits efficiently.
